# Silicon-Induced Morphological, Biochemical and Molecular Regulation in *Phoenix dactylifera* L. under Low-Temperature Stress

**DOI:** 10.3390/ijms24076036

**Published:** 2023-03-23

**Authors:** Saqib Bilal, Taimoor Khan, Sajjad Asaf, Nasir Ali Khan, Syed Saad Jan, Muhammad Imran, Ahmed Al-Rawahi, Abdul Latif Khan, In-Jung Lee, Ahmed Al-Harrasi

**Affiliations:** 1 Natural & Medical Sciences Research Center, University of Nizwa, Nizwa 616, Oman; 2 School of Life Sciences, University of Warwick, Coventry CV4 7AL, UK; 3 Department of Plant and Soil Science, Institute of Genomics for Crop Abiotic Stress Tolerance, Texas Tech University, Lubbock, TX 79409, USA; 4 Division of Plant Biosciences, School of Applied Biosciences, College of Agriculture & Life Science, Kyungpook National University, 80 Dahak-ro, Buk-gu, Daegu 41566, Republic of Korea; 5 Department of Engineering Technology, University of Houston, Sugar Land, TX 77479, USA

**Keywords:** oxidative stress, plant plasma membrane ATPase, salicylic acid, jasmonic acid, *inducer of CBF expression 1* (*ICE-1*), lipid peroxidation, catalase, low silicon gene

## Abstract

Climate changes abruptly affect optimum growth temperatures, leading to a negative influence on plant physiology and productivity. The present study aimed to investigate the extent of low-temperature stress effects on date palm growth and physiological indicators under the exogenous application of silicon (Si). Date palm seedlings were treated with Si (1.0 mM) and exposed to different temperature regimes (5, 15, and 30 °C). It was observed that the application of Si markedly improved fresh and dry biomass, photosynthetic pigments (chlorophyll and carotenoids), plant morphology, and relative water content by ameliorating low-temperature-induced oxidative stress. Low-temperature stress (5 and 15 °C), led to a substantial upregulation of ABA-signaling-related genes (*NCED-1* and *PyL-4*) in non Si treated plants, while Si treated plants revealed an antagonistic trend. However, jasmonic acid and salicylic acid accumulation were markedly elevated in Si treated plants under stress conditions (5 and 15 °C) in comparison with non Si treated plants. Interestingly, the upregulation of low temperature stress related plant plasma membrane ATPase (*PPMA3* and *PPMA4*) and short-chain dehydrogenases/reductases (*SDR*), responsible for cellular physiology, stomatal conductance and nutrient translocation under silicon applications, was observed in Si plants under stress conditions in comparison with non Si treated plants. Furthermore, a significant expression of *LSi-2* was detected in Si plants under stress, leading to the significant accumulation of Si in roots and shoots. In contrast, non Si plants demonstrated a low expression of *LSi-2* under stress conditions, and thereby, reduced level of Si accumulation were observed. Less accumulation of oxidative stress was evident from the expression of *superoxide dismutase (SOD)* and *catalase (CAT)*. Additionally, Si plants revealed a significant exudation of organic acids (succinic acid and citric acid) and nutrient accumulation (K and Mg) in roots and shoots. Furthermore, the application of Si led to substantial upregulation of the low temperature stress related soybean cold regulated gene (*SRC-2*) and *ICE-1* (*inducer of CBF expression 1*), involved in the expression of CBF/DREB (C-repeat binding factor/dehydration responsive element binding factor) gene family under stress conditions in comparison with non Si plants. The current research findings are crucial for exploring the impact on morpho-physio-biochemical attributes of date palms under low temperature and Si supplementation, which may provide an efficient strategy for growing plants in low-temperature fields.

## 1. Introduction

Plants have been subjected to numerous abiotic stresses caused by climate change, which are occurring at an alarming rate, resulting in the loss of natural habitats and decreased agricultural productivity [1]. Globally, abrupt environmental changes have threatened sustainable crop yield and raised concerns about food security [2,3]. Different kinds of abiotic stresses, including cold, heat, drought, salinity and heavy-metal contamination have been reported extensively and caused around 70% of global crop loss by hampering cellular, biochemical and physiological processes and thereby retarding the growth of plants [3,4]. Temperature as abiotic stress is responsible for both limiting plants’ productivity and their geographical distribution, thereby threatening overall crop development [4]. Beyond range, the lower or higher temperatures that demolish plant growth and survival are among the caustic environmental factors and are referred to as extreme temperature losses throughout the world. Plants require an optimum temperature for proper growth and resilience to stresses [5].

Low temperature is a detrimental environmental stress that significantly affects plant survival, as one among the extreme temperature stresses [6]. Low temperature as an abiotic stress is categorized into freezing stress (below 0 °C) and cold or chilling stress (below 20 °C) [6]. In California, cold stress has destroyed fruit trees over an area of more than 400,000 ha. At the same time, the United States have reported massive economic losses due to crop destruction caused solely by chilling stress, more than by any other abiotic stress [7]. The yield of wheat grains is predicted to decline by 1.6% for every degree change in temperature [8,9]. Considering the climate conditions, it is a particular challenge for scientists and plant breeders to cope with temperature fluctuations and devise new strategies to overcome the challenges induced by low-temperature stresses.

Low-temperature stress is also reported to inhibit the reproductive development of plants by hampering pollen production and the growth of pollen tubes, which subsequently disturbs the fertilization process [10]. Moreover, it induces wilting [11], chlorosis [12], reduced leaf expansion [13], damages reproductive components, and leads to necrosis [14,15]. Mechanistically, the necrosis of plant cells is induced by the excessive generation of reactive oxygen species (ROS) [16]. Low-temperature stress is also known to cause ice formation in extracellular spaces and to reduce the potential of apoplastic solution that makes water flow out of the cells; consequently, membrane integrity is damaged and electrolyte leakage is triggered [17,18]. Low-temperature stress is directly involved in negatively affecting cellular components and processes that ultimately retard the growth of plants. Physiologically, it has been acknowledged that it alters the rigidity of cellular membranes and results in a loss of membrane function along with disrupting macromolecules [19]. Recently, a study reported cell leakage and tissue damage in *Vitis vinifera* leaves exposed to freezing temperatures [20]. Similarly, low-temperature stress severely affects the photosynthetic system in plants, which further reduces yield and growth. Previous studies have reported the adverse impacts of low temperature on photosynthesis in plants of tropical and sub-tropical zones [21]. Other studies regarding low-temperature stress reported that it could constrain CO_2_ assimilation and was consistently found to reduce the photochemical efficiency of PS-II [22].

Silicon exists in ample amounts in the earth’s crust (28%), as a significant component of soil that is effectively supporting the growth and survival of crops [23,24]. It is not yet considered an essential nutrient but has proved to play an extensive plant-growth-promoting role, particularly in stress conditions [23,25]. The role of Si application in mitigating or neutralizing harmful effects has subsequently been disclosed for several stresses, including temperature [26], salinity and drought [25,27], heavy metals stress [28], and diseases in plants [29]. Additionally, it is also known for playing a vital role in improving nutrient deficiencies in plants, including potassium [30] and iron [31]. It can translocate into cell membranes of roots swiftly and further into leaves or aerial parts of plants via specified influx transporters of parenchyma cells of the xylem [32]. However, variation does exist in plants’ potential to accumulate Si, and subsequently contradictory observations have previously been reported. Based on its affinity for ions, it usually occurs as silicate, silica, and silicic acid [33]. It also has been identified as a significant component of the cell wall, where it functions in parallel to lignin as a resisting material in several plants [34]. Si promotes an adaptive response during stress by signaling transduction and activation of secondary metabolism to raise antioxidants, enhance hormonal activities, improve nutrients, and promote organic acid uptake as well as translocation in plants [35,36]. A meta-analysis showed more translocation in the leaf epidermis and a remarkably reduced sodium ion concentration in plant leaves under stress [37]. Recently, its role in defense-response-related signal transduction, including in the jasmonic acid (JA) pathway [38], as well as abscisic acid (ABA) and salicylic acid (SA) regulation [39,40] have been described.

*Phoenix dactylifera* L., locally known as date palm, serves as an integral and major fruit crop due to its high nutritional and economic features [24]. *Phoenix dactylifera* L. plays significant roles in the economy, society, and environment of countries in arid and semi-arid zones, including Oman. Its cultivation and production keep Oman in the 8th position among the largest global producers, for typical production values of >250,000 metric tons annually [2]. Among all fruits grown in Oman, it accounts for about 82%, taking up around 50% of the total cultivation area. However, the changing climate-induced environmental stresses have markedly affected its adaptation [41,42]. Despite the fact that the date palm can endure harsh environmental situations, its production and quality are negatively affected by several factors, including pests, diseases, and extensive exploitation [42]. Hence, the date palm requires serious attention to develop stress-alleviating strategies and improve its adaptive response. Previously, our research group has investigated silicon for stress alleviation in date palms against drought, salinity, and metal stresses, and found promising effects [2,24]. However, scientific reports are lacking regarding the lethal impacts of low-temperature stress on the date palm on the physiological, biochemical and molecular levels. Furthermore, the impact of silicon application on the date palm for the mitigation of low-temperature stress via modulation of metabolic activities, anatomical regulation, biochemical and molecular alteration is yet to be investigated. Therefore, the current study aims to unveil the effects of low-temperature stress and explore Si’s contribution to date palm defense. In particular, we aimed to investigate the underlying physiological, biochemical, and molecular mechanisms for low-temperature tolerance in the date palm and identify key factors responsible for stress tolerance. Therefore, we elucidated Si-induced hormonal (ABA, JA, SA) cross talk for regulating oxidative-stress-related enzymes, photosynthetic activities and macro nutrient uptake. Moreover, silicon transporter and low-temperature-responsive gene regulation for alleviating low-temperature stress were studied.

## 2. Results

### 2.1. Morphological and Physiological Impact of Silicon on Date Palm Seedlings under Low-Temperature Stress

**Figure 1:**
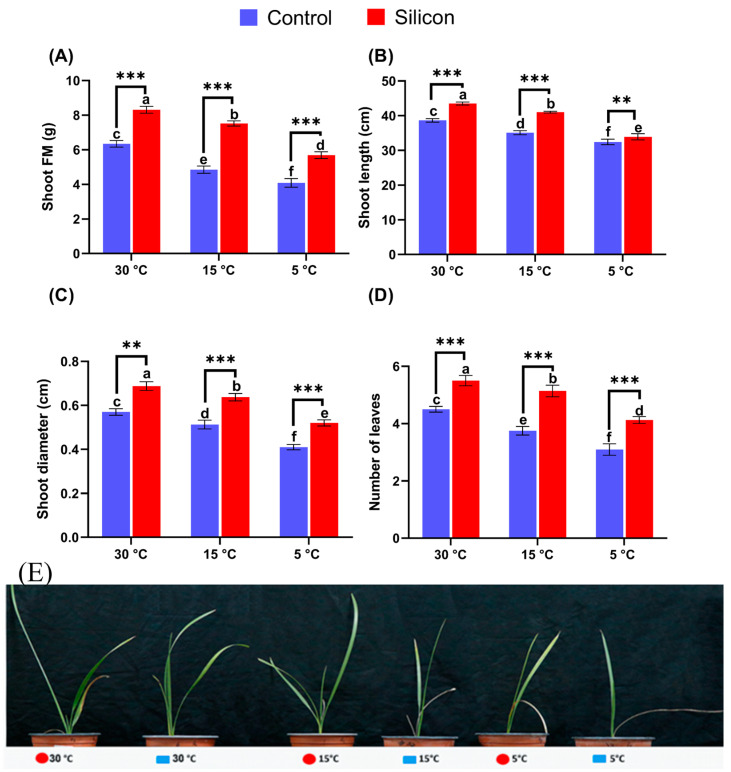
Influence of Silicon (Si) on growth attributes of date palm exposed to various low-temperature stress conditions (5, 15, and 30 °C) i.e., (**A**) Shoot FM (Fresh Mass), (**B**) Shoot Length, (**C**) Shoot diameter, (**D**) Number of leaves per plant and (**E**) Date palm seedling picture. Different symbols (small letters above bars) indicate that values are significantly different (*p* < 0.05). ** *p* < 0.01 between two relevant treatments, *** *p* < 0.001. Means were analyzed for finding significant differences among treatments by performing Duncan’s multiple range test and one-way ANOVA analysis of variance in GraphPad Prism (v7.01). Values represent means (8 replicates) ± standard error.

Effects of low-temperature stress had a detrimental impact on date palm growth and development, while silicon positively influenced its growth supporting potential by mitigating low-temperature stress. Leaves are directly exposed and possess more sensitivity to low temperature than other parts of the plant. The silicon supplementations significantly (*p* < 0.005) enhanced biomass and resulted in maximum fresh shoot mass (8.3 ± 0.15 g) at 30 °C followed by Si treated plants with 7.5 ± 0.05 and 5.6 ± 0.19 g under stress conditions (15 and 5 °C), respectively, in comparison with non Si treated plants (Figure 1A). Similarly, the shoot length was significantly enhanced (43.5 ± 1.9 cm) in Si treated plants at 30 °C, followed by Si treated plants in low-temperature stress conditions at 15 and 5 °C, respectively (Figure 1B). Moreover, Si treated plants exhibited a significantly improved diameter under normal conditions (30 °C) compared to that of non Si treated plants. The decrease in shoot diameter at 15 and 5 °C was significantly upregulated in Si treated plants by demonstrating an approximately 1.24- and 1.26-fold enhanced diameter compared to their respective treatments (Figure 1C). The number of leaves per plant was recorded to increase in plants with silicon treatments under normal conditions (30 °C) but was significantly reduced under stress conditions (15 and 5 °C) (Figure 1D). However, supplementation of Si markedly enhanced the number of leaves at 15 and 5 °C compared to their respective treatments. Henceforth, low-temperature stress considerably reduced the growth attributes of date palm shoots in lower-temperature treatments.

**Figure 2:**
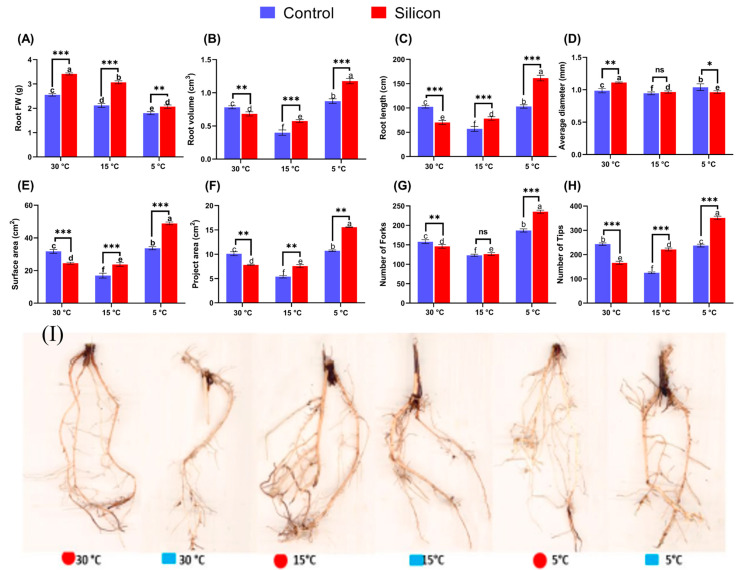
Influence of Silicon (Si) on root morphology and developmental architecture of date palm exposed to various low-temperature stress conditions (5, 15 and 30 °C), (**A**) Root Fresh Mass, (**B**) Root Volume, (**C**) Root length, (**D**) Root diameter, (**E**) Surface area, (**F**) Project area, (**G**) Number of Forks, and (**H**) Number of Tips, analyzed by WinRHIZO image analysis system (Regent Instruments, Inc., Sainte-Foy, QC, Canada). (**I**) Date palm root picture. Different symbols (small letters above bars) indicate that values are significantly different (*p* < 0.05). * *p* < 0.05 between two relevant treatments, ** *p* < 0.01, *** *p* < 0.001, ns: not significant. Means were analyzed for finding significant differences among treatments by performing Duncan’s multiple range test and one-way ANOVA analysis of variance in GraphPad Prism (v7.01). Values represent means (8 replicates) ± standard error.

Likewise, the root samples from all treatments were analyzed for phenotypic data. The findings revealed that Si significantly aided growth attributes by developing an overall root architecture, particularly under low-temperature stress conditions. The average fresh mass of roots with Si supplementation was found to be significantly higher in controls (30 °C) than in roots exposed to low-temperature stress with or without silicon treatments (Figure 2A). At the same time, the Si application resulted in a significant enhancement in root volume and root length under stress conditions and the highest values were recorded (1.175 ± 0.05 cm and 161.5 ± 0.83 cm, respectively) at 5 °C compared to control (Figure 2B,C). The average diameter of roots was slightly increased by silicon supplementation in normal conditions (30 °C) and decreased at 5 °C conditions as compared with non-treated roots (Figure 2D). Similarly, the root surface and project areas of Si treated plants were reduced under normal conditions (30 °C) in comparison with non Si treated plants, but markedly increased at 15 °C and 5 °C stress in Si treated plants compared to non-treated plants. Moreover, severe low temperature stress induced fork and tip formation in plants exposed to low-temperature stress (Figure 2G,H) was observed. However, Si supplementation led to the highest value of tip and fork formation in roots at 5 °C stress in demonstrating approximately 1.2- and 1.5-fold enhancements, respectively, in comparison with the relative treatments. Hence, the morphological and physiological data for shoots and roots show that low-temperature stress considerably reduced the overall growth of date palms. However, silicon-treated plants were conclusively found to defend their growth more under low-temperature stress compared to plants under normal conditions and without silicon application.

### 2.2. Influence of Silicon on Photosynthetic Pigments under Low-Temperature Stress

**Figure 3:**
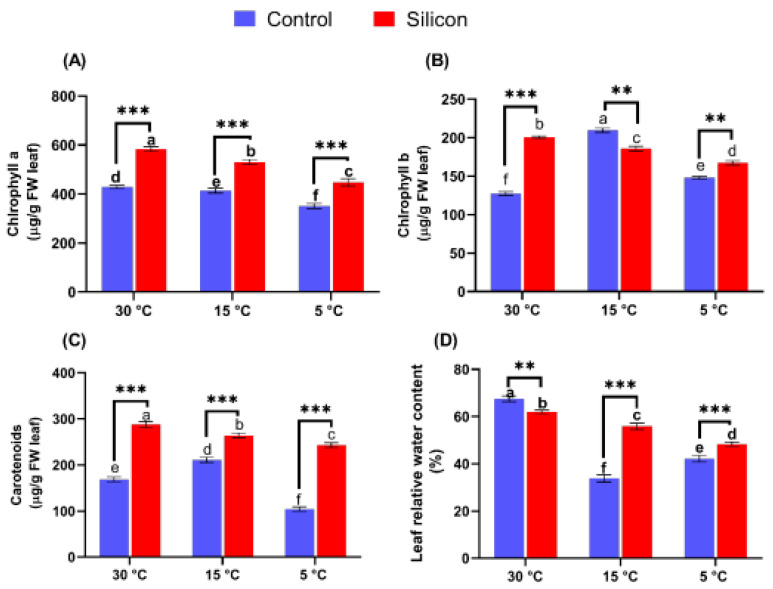
Accumulation of photosynthetic pigments; (**A**) Chlorophyll a, (**B**) Chlorophyll b, (**C**) Carotenoids, and (**D**) Relative Water status in date palms under interactive influence of exogenously applied and low-temperature stress conditions (5, 15 and 30 °C). Different symbols (small letters above bars) show significant differences among values (*p* < 0.05). ** *p* < 0.01 between two relevant treatments, *** *p* < 0.001. Means for all treatment values were used to determine significant differences by Duncan’s multiple range test and performing one-way ANOVA analysis of variance in GraphPad Prism (v7.01). Values represent means (of 8 replicates) ± standard error.

To further investigate the negative effects of low-temperature stress and silicon treatment in date palms, we also investigated chlorophyll a, b and carotenoids along with relative water contents in shoots. Plants treated with Si had raised levels of chlorophyll a and b in shoots exposed to 30 °C by approximately 35% and 57% in comparison with non Si treated plants, respectively. Exposure to low-temperature stress reduced the accumulation of Chl a and Chl b. However, Si treated plants maintained significantly higher levels of Chl a at both 15 and 5 °C by 28% and 27%, and Chl b by 14% at 5 °C in comparison with non Si treated plants, respectively. Following a similar trend, Si supplemented plants exhibited significantly (*p* < 0.005) enhanced levels of carotenoid accumulation at 30 °C, followed by 15 and 5 °C, respectively, compared to non Si treated plants (Figure 3A–C). The stress alleviating response of plants supplemented with Si or without silicon was further determined via measuring their relative water content (RWC). The results revealed that maximum RWC was found in non Si treated plants at 30 °C compared to Si treated plants (Figure 3D). However, the silicon was found to significantly retain the RWC potential of plants under low-temperature stress conditions, by demonstrating around 1.6- and 1.2-fold higher levels in comparison with non Si treated plants, respectively. Overall, silicon augmented chlorophyll and water content, which successively support the photosynthetic process and retain plant growth under low-temperature stress.

### 2.3. Silicon Influences Anti-Oxidative System of Date Palm Seedlings under Low-Temperature Stress

**Figure 4:**
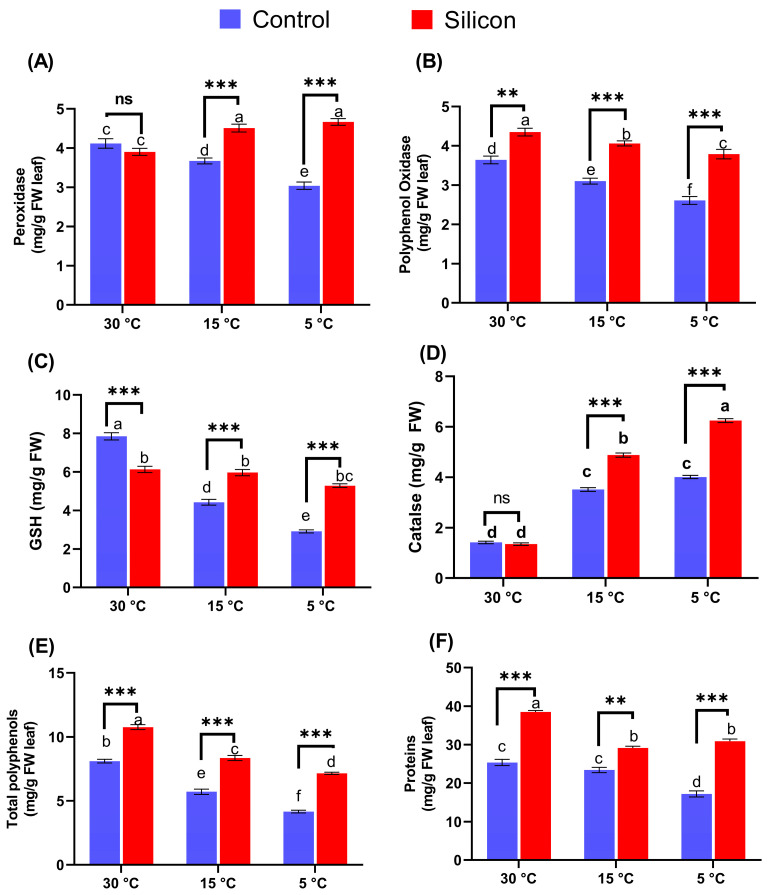
The influence of exogenous silicon (Si) on Anti-oxidants; (**A**) Peroxidase (POD), (**B**) Polyphenol oxidase (PPO), (**C**) Reduced glutathione (GSH), (**D**) Catalase (CAT), (**E**) Polyphenol (PP), and (**F**) Protein contents in date palms under low-temperature stress conditions (5, 15 and 30 °C). Means for all respective treatments were used to determine significant differences (*p* > 0.05) via Duncan’s multiple range test and performing one-way ANOVA analysis of variance in GraphPad Prism (v7.01). Symbols (small letters above bars) show significant differences and values represent mean (of 6 replicates) ± standard error. ** *p* < 0.01 between two relevant treatments, *** *p* < 0.001, ns: not significant.

The effects of Si supplementation were further evaluated by examining the regulation of antioxidant enzyme activities for countering the adverse impacts of stress. The current findings reveal that POD differences between Si treated and non-treated plants were found to be insignificant under control conditions (30 °C), whereas the stress conditions led to the alteration of POD activity in plants. The significant effects of silicon on date palm plants resulted in a marked accumulation of POD activity, 1.5- and 1.2-fold at 5 and 15 °C in comparison with respective treatments (Figure 4A), respectively. Similarly, PPO activity showed significant variation (*p* < 0.05) in plants supplemented with or without silicon under control conditions. PPO activity was also significantly (*p <* 0.005) reduced in non Si treated plants subjected to low-temperature stress at 5 and 15 °C (Figure 4B). However, the deleterious impacts of stress conditions were substantially reduced in Si treated plants, as evidenced by a 1.4-times and 1.3-times higher PPO level at 5 and 15 °C in comparison with the respective non Si treatments, respectively. Similarly, reduced glutathione reductase (GSH) activity was also found, and results showed that Si treated plants had a significantly lower level of GSH activity under control conditions (30 °C) compared to non Si treated plants. However, 5 and 15 °C stress markedly downregulated GSH activity of non Si treated plants in comparison with the respective Si treatments, approximately 1.8- and 1.3-fold, respectively (Figure 4C). Moreover, catalase (CAT) under control conditions (30 °C) was found to be insignificantly different between Si treated and non-treated plants and significantly lower than for other treatments (Figure 4D). However, exposure to stress conditions enhanced the level of CAT activity in all treatments, and Si application resulted in around 1.5- and 1.4-fold higher activities compared to the respective non Si treated plants at 5 and 15 °C, respectively (Figure 4D).

Moreover, the total polyphenol (TPP) and protein contents were also determined. The polyphenol production was significantly increased by Si in plants under control conditions (30 °C), with a 1.3-times higher level compared to non Si treated plants (Figure 4E). In stress conditions, the accumulation of TPP was significantly downregulated in non Si treated plants by 45% and 73% when compared with the respective treatments of Si treated plants under 15 and 5 °C, respectively. Likewise, increased induction of protein content through silicon application was found. Under stress conditions (15 and 5 °C), Si treated plants exhibited an insignificant accumulation of proteins content compared to each other. However, Si treated plants in comparison with non Si treated plants under stress conditions showed significantly increased levels, with 1.2- and 1.7-fold, respectively.

### 2.4. Silicon Inhibits Lipid Peroxidation and O_2_^•–^ Generation under Low-Temperature Stress

**Figure 5:**
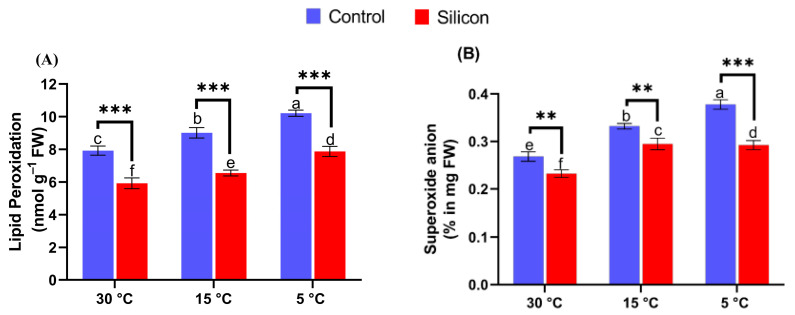
Stress alleviating effects of silicon (Si) exogenously applied on (**A**) Lipid peroxidation (MDA), and (**B**) Superoxide anion in date palms under low-temperature stress conditions (5, 15 and 30 °C) i.e., Means for all respective treatments were used to determine significant differences (*p* > 0.05) via using one-way (ANOVA), followed by Duncan’s multiple range test. Symbols (small letters above bars) show significant differences and values represent mean (of 6 replicates) ± standard error. ** *p* < 0.01 between two relevant treatments, *** *p* < 0.001.

In the current study, shoots samples directly exposed to low-temperature stress were screened for O_2_^•−^ and MDA assays to determine low temperature stress induced ROS generation. Results show that significant levels (*p* < 0.005) of O_2_^•–^ were observed in plants without Si treatment upon exposure to low-temperature stress conditions (5 °C) in comparison with other treatments (Figure 5A). Nevertheless, the detrimental effects of low-temperature stress were markedly reduced by Si application in comparison with non Si treatments. The O_2_^•−^ level was reduced significantly by Si application under increased low-temperature stress (5 °C), 1.29-fold, and 1.12-fold under 15 °C stress in comparison with their respective treatments.

Similarly, the peroxidation of membrane-associated lipids induced by low-temperature stress was also assessed by measuring levels of MDA contents. Significant levels of MDA accumulation were recorded in non-treated plants exposed to low-temperature stress. However, the silicon treatments significantly reduced MDA levels in control and stress conditions compared with non-treated plants (Figure 5B). For instance, the levels of MDA under control conditions (30 °C) were detected to be downregulated by 33.8% in Si treated plants as compared to non Si treated plants. In contrast, the plant stress conditions (15 °C and 5 °C) were found to have more lipid peroxidation by exhibiting significant levels of MDA in comparison with treatments of control conditions. The degree of lipid peroxidation was markedly reduced by Si treatments under stress conditions, and the MDA level was significantly reduced, by 38.4% and 30.8% at 15 °C and at 5 °C, respectively, compared to non Si treated plants.

### 2.5. Determination of Endogenous Hormones

**Figure 6:**
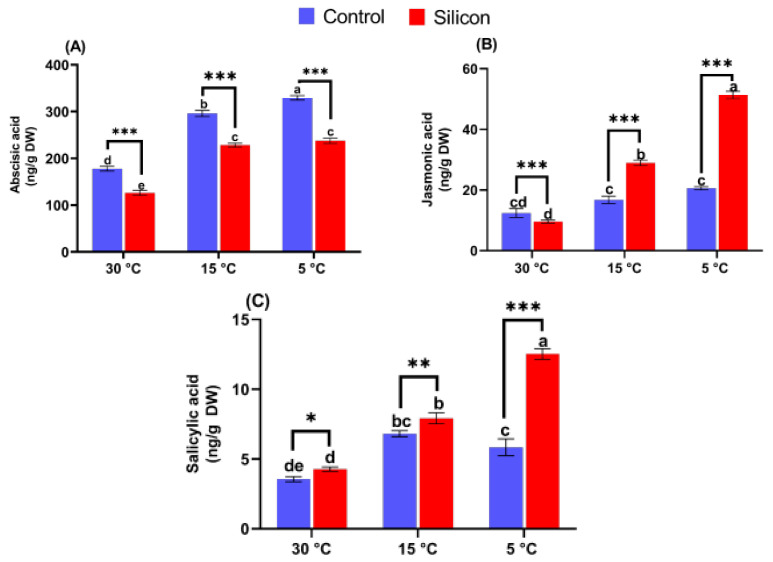
Effects of low-temperature stress regimes (5, 15 and 30 °C) and exogenous Silicon (Si) on regulation of endogenous hormones; (**A**) Abscisic acid, (**B**) Jasmonic acid, (**C**) Salicylic acid of date palm seedlings. Data represented as mean (6 replicas) ± standard error, symbols (small letters above bars) denote significant differences among values as evaluated by using one-way (ANOVA), followed by Duncan’s multiple range test. * *p* < 0.05 between two relevant treatments, ** *p* < 0.01, *** *p* < 0.001.

The regulation of signal transduction pathways by silicon-induced endogenous phytohormonal cross talk was studied to unveil the beneficial effects of Si treatments under low-temperature stress. Current results show that under control conditions (30 °C), Si treated plants followed by non Si treated plants presented the lowest accumulation of ABA compared to other treatments (Figure 6A). In contrast, low-temperature stress significantly (*p* < 0.005) stimulated the accumulation of endogenous ABA in plants when compared with the plants of control conditions. However, the alleviated level of ABA was drastically reduced in Si treated plants, 1.3- and 1.4-fold, when compared to the respective non Si treated plants at 15 °C and 5 °C. In addition, the endogenous jasmonic acid level was also determined under different temperature regimes. A lower level of JA was observed in plants treated with silicon under control conditions (30 °C), while higher levels of JA production in Si treated plants under stress conditions were detected (Figure 6B). With a decrease in temperature, Si treatment led to an increase in JA production in showing approximately 1.7- and 2.5-fold higher levels at 15 °C and 5 °C compared to the respective treatments. Furthermore, endogenous SA accumulation was detected to be insignificantly different between Si treated plants and non Si treated plants under control conditions (Figure 6C). However, a slight enhancement was detected in Si treated plants at 15 °C in comparison with non Si treated plants. A further decrease in temperature (5 °C) led to a significant (*p* < 0.005) down-regulation of SA content in non Si treated plants, while Si treated plants exhibited an approximately 47% higher accumulation compared to non Si treated plants.

### 2.6. Determination of Organic Acid Levels

**Figure 7:**
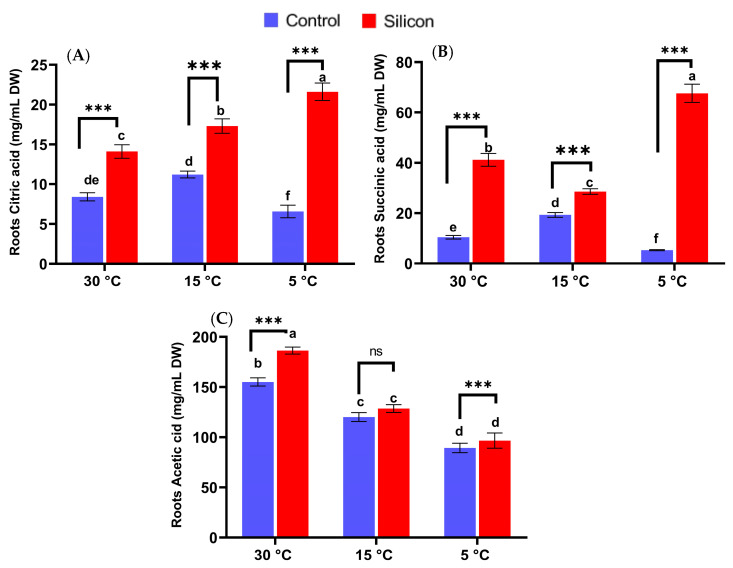
Effects of low-temperature stress regimes (5, 15 and 30 °C) and exogenous Silicon (Si) on regulation on organic acids regulation; (**A**) Citric acid, (**B**) Succinic acid, (**C**) Acetic acid of date palm roots. Data represented as mean (6 replicas) ± standard error, symbols (small letters above bars) denote significant differences among values as determined by Duncan’s multiple range test. *** *p* < 0.001 between two relevant treatment, ns: not significant.

Date palm plants under low-temperature stress were evaluated for quantifying organic acids to assess the effect of Si application on their regulation (Figure 7). The current findings show that supplementation of Si under control conditions led to a significant induction of citric acid, succinic acid and acetic acid in date palms in comparison with non Si treated plants. However, the induction of citric acid and succinic acid in the 5 °C stress condition was found to be substantially enhanced in Si treated plants compared to other treatments. For instance, Si treated plants at 5 °C showed approximately a 3.3- and 2.0-fold higher level of citric acid and a 12.7- and 3.5-fold higher level of succinic acid in comparison with non Si treated plants at 5 °C and 15 °C, respectively (Figure 7A,B). In contrast, no significant variation under stress conditions at 15 °C and 5 °C was detected between Si treated plants and non Si treated plants. The level of citric acid revealed no significant impacts of Si treatments on date palms under stress conditions in comparison with non-treated plants.

### 2.7. Influence of Si on Date Palm Morphology under Stress

**Figure 8:**
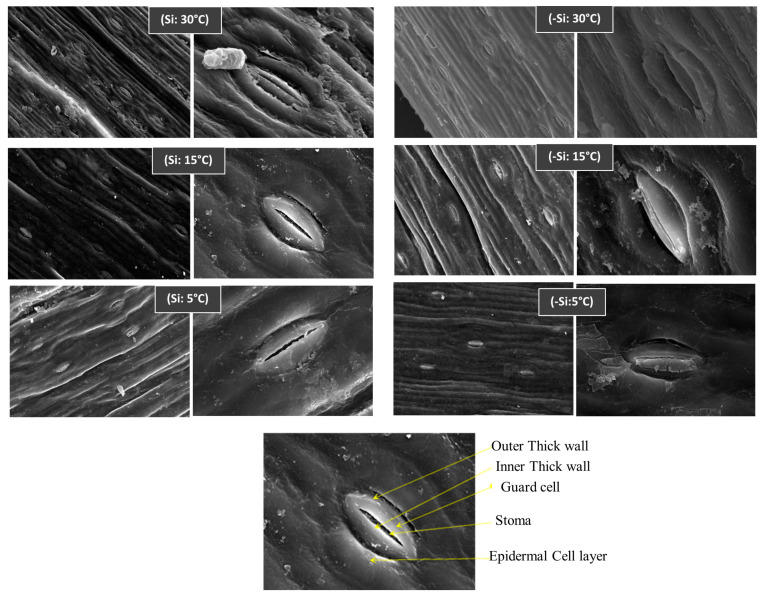
Influence of Silicon (Si) on stomatal apertures and conductance in date palms under low-temperature stress conditions (5, 15 and 30 °C), Si treated (Si) or non-treated (-Si). Three biological replicates for each sample were screened with a Scanning Electron Microscope (SEM), at magnification 3000×, scale bar = 5 µm.

Leaves are directly exposed to low-temperature environmental stress, and therefore, date palm leaves treated with silicon were screened with SEM after low-temperature exposure. Low-temperature stress exerted remarkable effects on the cellular morphology of guard and epidermal cells, resulting in decreased stomatal conductance that can be seen by the size of stomata in non-treated plants. Contrarily, the stomatal apertures and epidermal cells were observed to be morphologically maintained in silicon-treated plants under normal or stressed conditions (Figure 8). Silicon also enhanced stomatal conductance under stressed conditions compared to non-treated plants, resulting in osmotic homeostasis.

**Figure 9:**
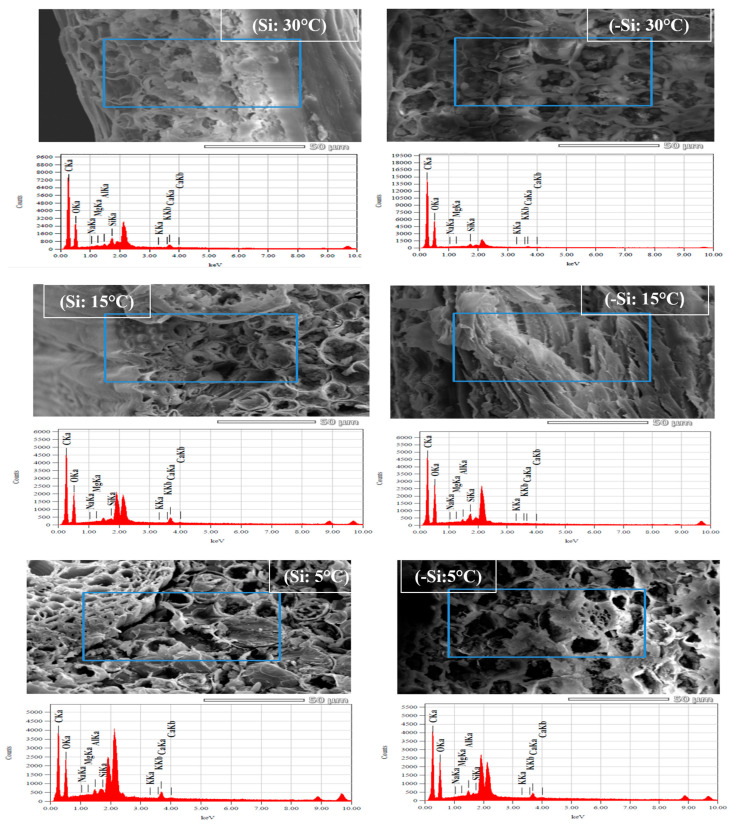
SEM-EDS analysis of date palm leaves treated with silicon (Si) and non-treated (-Si) under low-temperature stress conditions (5, 15 and 30 °C). The highlighted areas (in blue) were chosen for EDS-based elemental analysis; graphs show EDS-elemental peaks.

Furthermore, the corresponding EDX-elemental analysis of leave samples showed the interference of silicon deposition in treated and non-treated samples (Figure 9). Overall, EDS spectra showed the presence of not only Si but also Mg, Ca, K and sodium Na (Figure 9).

### 2.8. ICP-Elemental Analysis for Silicon (Si) and Nutrients Accumulation

**Figure 10:**
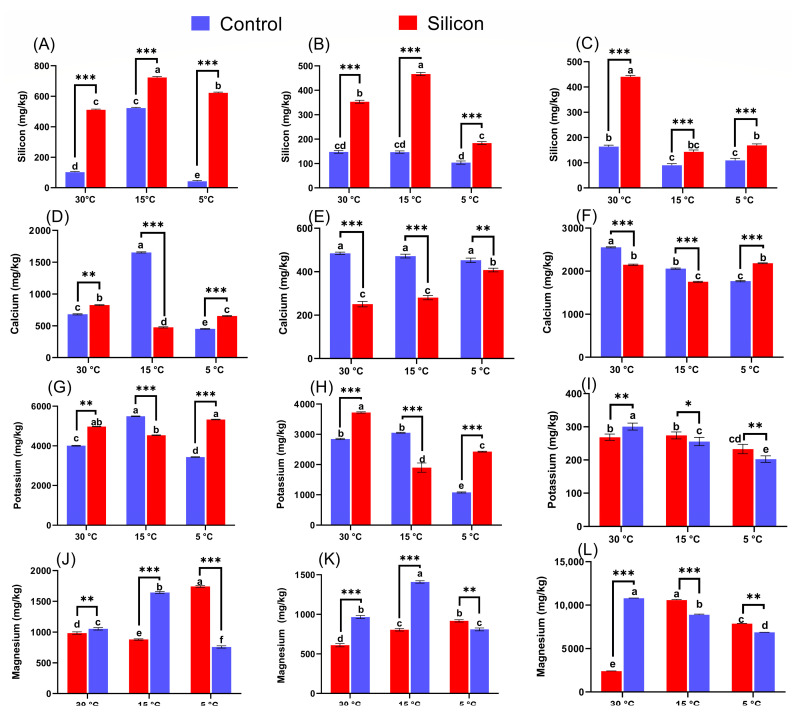
ICP-elemental analysis for Silicon (Si) and nutrients regulation in (**A**,**D**,**G**,**J**) shoot, (**B**,**E**,**H**,**K**) root of date palm, and soil (**C**,**F**,**I**,**L**), respectively, under low-temperature stress conditions (5, 15 and 30 °C). Different symbols (small letters above bars) show that the values are significantly different (*p* < 0.05) according to Duncan’s multiple range test and one-way ANOVA in GraphPad Prism (v7.01). * *p* < 0.05 between two relevant treatments, ** *p* < 0.01, *** *p* < 0.001. Values represent means (8 replicates) ± standard error.

Accumulation and translocation of Si as well as essential nutrients including Ca, K, and Mg were analyzed in shoots, roots, and soil to understand the impacts of Si application on plant-physiological functions under control and low-temperature stress conditions (Figure 10). The accumulation of Si was significantly higher in Si treated plants than in non Si treated plants. Meanwhile, the accumulation of Si was recorded to have decreased more in non Si treated plants at both 15 and 5 °C stress, 1.4- and 13.8-fold in shoots and 3.1- and 1.7-fold in roots when compared to other Si treatments. Moreover, a significant abundance of Si content was observed in the rhizospheric soil of Si treatment at 30 °C, equally followed by Si treatments at 15 and 5 °C compared to the relative non Si treatments. Similarly, Ca content was recorded as being enhanced in shoots and decreased in roots of Si treated plants under control conditions (30 °C) in comparison with non Si treated plants. In contrast, Si supplemented plants had significantly reduced Ca content in shoots at 15 °C and roots at both 15 and 5 °C stress conditions, respectively, in comparison with relevant non Si treatments (Figure 9). For instance, Si supplemented plants exhibited an approximately 3.5-fold lower Ca content in shoots at 15 °C and 1.6- and 1.1-fold lower in roots at 5 and 5 °C stress conditions in comparison with the relative non Si treatments. In addition, the Ca content of rhizospheric soil was markedly higher in non Si treatments under control conditions at 15 °C and significantly lower at 5 °C than for the respective Si treatments. Furthermore, the level of K was recorded to be substantially increased by supplementation of Si at control conditions and 5 °C stress conditions in both shoots and roots compared to non Si treatments, while the opposite trend was noticed in Si treated plants under 15 °C stress conditions. The level of Mg was thoroughly enhanced in the roots and shoots of non Si treatments at control conditions and 15 °C stress conditions when compared with the respective Si treatments. However, 5 °C stress drastically reduced the Mg contents of shoots and roots in non Si treatments compared to Si treatments, 2.2- and 1.1-fold, respectively. The content of K and Mg in rhizospheric soil exhibited significantly reduced levels in comparison with non Si treatments in control conditions. However, Si supplementation under stress conditions considerably increased K and Si levels in rhizospheric soil at both 15 and 5 °C stress conditions when compared with the comparative non Si treatments (Figure 9).

### 2.9. Si influences the Regulation of Abiotic Stress-Associated Genes

**Figure 11:**
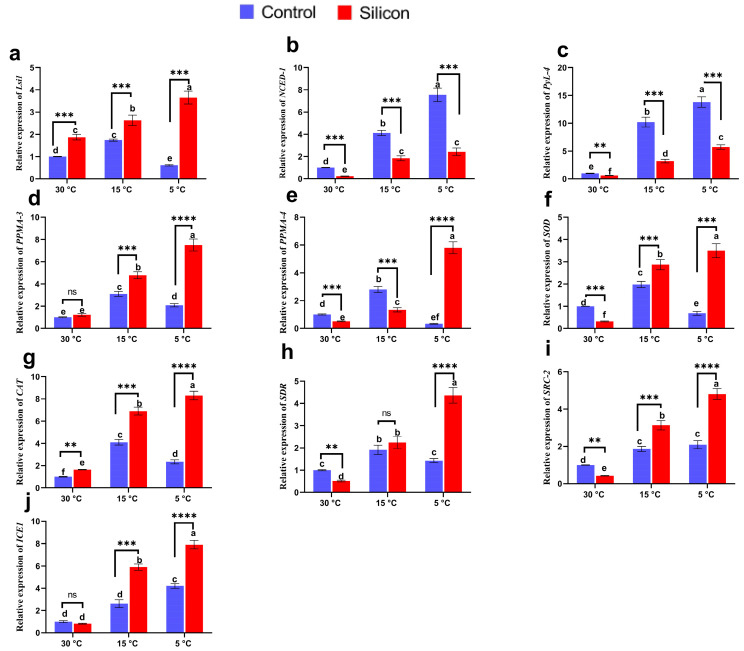
Influence of Silicon on expression pattern of abiotic stress-related genes in date palm seedlings under low-temperature stress (5, 15 and 30 °C). (**a**) *Low silicon 2 gene* (*Lsi2*), (**b**) *9-cis-epoxycarotenoid dioxygenase-1 gene* (*NCED-1*), (**c**) *pyrabactin resistance like gene* (*PyL-4),* (**d**,**e**) plasma membrane ATPase gene (*PMMA-3*, *PMMA-4)*, (**f**) *Superoxide dismutase* (SOD), (**g**) catalase (*CAT*), (**h**) *short-chain dehydrogenases/reductases* (*SDR*), (**i**) *soybean gene regulated by cold-2* (*SRC-2*), (**j**) *inducer of CBF expression 1* (*ICE1*). Data represented as mean ± standard error. The symbols (small letters above bars) denoted significant differences among treatments evaluated by Duncan’s multiple range test and multiple comparison *t*-test in GraphPad. ** *p* < 0.01 between two relevant treatments, *** *p* < 0.001, **** *p* < 0.0001, ns: not significant.

A molecular investigation of abiotic stress-related genes and their alteration under low-temperature stress and silicon application was performed (Figure 11). The silicon transporting gene (*Lsi-2*) was recorded to have significantly higher expression levels in Si treated plants at control conditions (30 °C) and stress conditions (15 and 5 °C). However, with the increase in stress severity, the expression level of Si treatments was enhanced, while non Si treated plants exhibited significantly reduced expression at higher levels (5 °C) of low-temperature stress. Moreover, ABA responsive gene (*NCED1* and *PyL4*) expression was significantly affected depending on the severity of low-temperature stress. The expression rates of *NCED1* and *PyL4* in non Si treated plants were significantly increased, 2.4- and 3.1-fold as well as 3.2- and 2.4-fold at 15 and 5 °C, respectively, in comparison with Si treated plants. Furthermore, the plasma membrane is considered to be the major site for responding to temperature stresses. Therefore, the transcript accumulation of plant plasma membrane ATPase (*PPMA-3* and *PPMA-4*) was determined. The current results reveal that under control conditions, Si treatments showed an insignificant alteration of *PPMA-3* and slightly reduced expression of *PPMA-4* compared to non Si treatments. However, exposure to low-temperature stress significantly downregulated the transcript accumulation of *PPMA-3* in non Si treated plants, showing approximately 1.5- and 3.6-times lower levels in comparison with Si treated plants at 15 and 5 °C, respectively. In contrast, Si treated plants exhibited a significantly lower level of *PPMA-4* expression at 15 °C and significantly elevated level of expression at 5 °C compared to non Si treated plants. Furthermore, oxidative-stress-related gene expression demonstrated that Si supplementation downregulated *SOD* and upregulated *CAT* under control conditions in comparison with non Si treated plants. However, the induction of low-temperature stress significantly enhanced *CAT* and *SOD* expression levels at 15 °C in non Si treated plants in comparison with non Si treated plants under control conditions. Moreover, the transcript accumulation of these genes was significantly downregulated in non Si treated plants at 5 °C stress and markedly enhanced in Si supplemented plants, 5.14- and 3.7-fold, respectively, in comparison with non Si treated plants. Likewise, transcript accumulation of the *short-chain dehydrogenases/reductases* (*SDR*) gene was found to be insignificantly different between Si and non Si treated plants at 15 °C stress. Contrarily, the expression level of *SDR* was substantially downregulated at 5 °C stress in non Si treated plants and markedly enhanced, 3.1-fold, in Si treated plants compared to Si treated plants. Additionally, low temperature stress related gene expression revealed that Si supplementation markedly induced the transcript accumulation of the homolog of the *SRC2* (soybean gene regulated by cold-2) and *ICE-1 (inducer of CBF expression 1)* genes under both regimes (15 and 5 °C) of low-temperature stress in comparison with non Si supplemented plants.

### 2.10. Histogram-Correlation Analysis and Principal Component Analysis (PCA)

**Figure 12:**
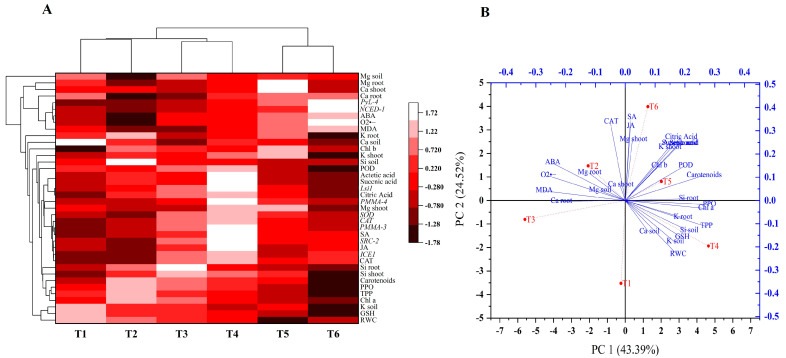
(**A**) Heatmap histogram correlation analysis between different analyzed attributes of Si and non Si treated palms under stress conditions. (**B**) Pearson correlation among different physiological and biochemical traits of date palms studied in the current experiment. The different abbreviation used in the analysis are as follows: magnesium (Mg), Calcium (Ca), pyrabactin resistance like gene (*PyL-4)*, 9-cis-epoxycarotenoid dioxygenase-1 gene (*NCED-1*), abscisic acid (ABA), superoxide anion (O2^•−^), Malondialdehyde (MDA), Potassium K, chlorophyl a and b (Chl a,b), Silicon (Si), Low silicon 2 gene (*Lsi2*), plasma membrane ATPase gene (*PMMA-3*, *PMMA-4)*, superoxide dismutase gene (*SOD*), *catalase* gene (*CAT*), salicylic acid (SA), soybean gene regulated by cold-2 (*SRC-2*), jasmonic acid (JA), inducer of CBF expression 1 (ICE1), polyphenol peroxidase (PPO), total polyphenols (TPP), peroxidase (POD), Glutathione reductase (GSH), relative water content (RWC). The different treatments of the study are described as T1 (non Si plants at 30 °C), T2 (non Si plants at 15 °C), T3 (non Si plants at 5 °C), T4, (Si plants at 30 °C), T5 (Si plants at 15 °C), T6 (Si plants at 5 °C).

The histogram of Pearson correlation analysis is shown to determine a correlation between the studied attributes (physiological, biochemical and molecular) of date palms under control and low-temperature stress conditions with and without supplementation of Si (Figure 12). Significant variation was noticed in organic acids, *LSi-2*, hormonal modulation (JA, SA) and the soil nutrient regulation of plants treated with or without Si under control conditions (30 °C). Moreover, histograms clearly show significant differences under higher levels of low-temperature stress (5 °C) in Si and non Si spiked plants, particularly in terms of ABA, *PyL-4*, *NCED-1* oxidative stress generation, POD, GSH and Si accumulation in roots and shoots. The histogram illustrate a substantial degree of variation in the studied attributes of Si treated plants and non Si treated plants under stress conditions, specifically at 5 °C. Additionally, a PCA biplot analysis was carried out for physiological and biochemicals attributes, and eight treatment groups were assessed simultaneously to evaluate the effects of different levels of low-temperature stress with or without Si supplementation, as illustrated in Figure 12. PCA analysis revealed that the scattering of all components in the dataset provided a strong indication that low-temperature stress markedly affected different physiological and biochemical attributes in all treatments, with or without application of Si. The current findings show that CAT, MDA, O_2_^•–^ and uptake of Mg by roots and regulation in soil, as well as ABA and Ca uptake in shoots and roots, were negatively corelated with all other parameters in the database. Moreover, only the 15 °C and 5 °C stress treatments (T2 and T3) were distinctively separated from Si supplemented treatments (T5 and T6) under stress conditions, which demonstrates that Si supplementation had ameliorative effects on plants under low-temperature stress. The variables available in the same quadrant and not apart from each other were positively correlated.

## 3. Discussion

Nature nurtures every organism with a distinct provision of all suitable resources, including water, air, light, and temperature. Their excessive application induces physiological and biochemical stress on organisms and leads to abnormal growth. The extent and period of exposure to low-temperature stress are the key factors that determine the damage caused by low-temperature stress along developmental stages of the plant. Plants at the seedling stage are considered more susceptible to hostile conditions and are severely affected by low-temperature stress [43]. In the current study, we observed that the similar damaging effects of low-temperature stress resulted in reduced growth attributes as well as deteriorated date palm seeding morphologically by inducing a sunken, yellowish, and dry appearance under low-temperature stress, as previously reported by [44]. The present study revealed positive effects of Si application to plant seedlings by overcoming the detrimental effects of low-temperature stress by modulating plant biomass, photosynthetic components, the regulation of stomatal conductance as well as nutrient uptakes and translocation. Moreover, Si treatment had a positive impact on the modulation of ABA accumulation and signaling-related gene expression. Further, low-temperature induced ROS was substantially encountered by activation antioxidative system in Si plants, and demonstrated a higher expression of low temperature stress related genes and the accumulation of endogenous JA and SA. Mechanistically, low-temperature stress induces dehydration due to ice formation in intracellular spaces and leads to stomatal closure, and subsequently decreases membrane fluidity by affecting lipid membrane composition [45]. However, the exogenously applied Si in the current study was found to successfully maintain water content by regulating root architecture and stomatal conductance. In silicon-treated plants, stomatal conductance and apertures were also observed to be intact and improved. This could be ascribed to the silicon’s role in sustaining water levels by reducing transpiration rate through controlled stomatal and cuticular performance [46]. Moreover, low-temperature stress also inhibits photosynthetic activities, thereby declining growth and productivity. The photosynthetic pigments of date palm seedlings were remarkably improved by silicon under normal or low-temperature stress, whereas low-temperature stress dramatically degraded Chl a, Chl b, and carotenoids in non-treated plants. This could be attributed to higher levels of reactive oxygen species, which degrade photosynthetic pigments, as well as lower enzymatic antioxidant activity [47,48]**.** The improved contents of carotenoids and chlorophyll in silicon-treated plants compared to non-treated plants demonstrate the stress-ameliorating effects of silicon application. Furthermore, the water content of date palm seedlings without Si treatments was significantly reduced under low-temperature stress in comparison with Si treated plants. This maintenance of RWC in Si treated plants may be ascribed to the stomatal opening and improved roots system, including root length, volume as well as the number of forks and tips to uptake and transport water under stress conditions.

Moreover, low-temperature stress also triggered oxidative stress generation in non Si date palm seedlings by accumulating H_2_O_2_ and O_2_^•–^. In contrast, Si treated date palm seedlings showed significantly higher accumulations of H_2_O_2_ and O_2_^•–^, which could be attributed to an upregulation of *SOD*, *CAT* genes, and therefore, marked activities of antioxidants (PPO, POD, CAT, GSH) were detected in order to combat low-temperature-induced oxidative stress [49]. Low-temperature stress also induces solute precipitation and the denaturation of proteins as an outcome of dehydration [49,50]. We found a drastic decrease in the concentration of soluble proteins, linked with the reduced growth of non-treated date palm seedlings under low-temperature stress compared with non Si treated plants. Under abiotic stresses, different proteins are employed by the date palm to sustain osmoregulation and redox regulation, signal transduction, photosynthesis and secondary metabolism [51]. However, the significant reduction in total soluble proteins of non Si plants could be due to the generation of higher levels of ROS, which possibly destroy cellular membranes involved in amino acid and protein formation; consequently, protein synthesis is reduced [52]. Therefore, the significantly increased levels of soluble proteins in Si treated plants under low-temperature stress suggest the stress-alleviating impacts of Si on date palms, since Si is positively reported for its active involvement in regulating mRNA and plays vital role in binding amino acids for the formation of specific proteins [52]. Moreover, the study unveiled, based on histogram analysis, that an accumulation of Si in roots and shoots, MDA, O_2_^•–^ chlorophyll contents, ABA and carotenoids of non Si plants (T2 and T3) under low-temperature stress had a negative correlation with those of Si plants (Figure 12A). Moreover, PCA analysis also confirmed that Si in roots and shoots, photosynthetic pigments, organic acids (citric acid and succinic acid, POD, PPO, GSH) were combined in a positive trend and strongly linked with T5, T6 and T4, and other parameters were combined in a negative trend. Furthermore, plant groups (T4, T5, and T6) with Si treatment under normal and stress conditions were more separated from those with non Si treatments (T1, T2, and T3), which points out that Si application helped to significantly overcome the detrimental effects of low-temperature stress on the growth and biochemical attributes of date palm seedlings.

In plants, responses to abiotic stress are also arbitrated by phytohormonal signaling cascades that lead from sensing signals to stress-related gene expression. Phytohormones influence the generation and detoxification of excessive ROS generation due to abiotic stresses [53,54]. Silicon has recently been reported to induce a defense response by activating several regulatory and signaling pathways [38] and to confer abiotic stress resistance by maintaining homeostasis and up- or downregulating endogenous phytohormones [39,40]. Herein, we found variable levels of phytohormones (ABA, SA, JA) screened in date palms with or without silicon applications under low-temperature stress. ABA is a vital phytohormone with a significant role in plants’ life cycle and abiotic stress responses, and is usually upregulated under abiotic stress and induces stress resistance genetic expression [55,56]. However, a rapid increase in ABA levels under abiotic stresses can lead to the regulation of ABA biosynthesis genes, induce senescence of leaves and deterioration of the photosynthesis system and stomatal closure, all of which impede plant growth and development. Surprisingly, supplementation of Si under low-temperature stress significantly downregulated the level of ABA in date palm seedlings, implying that Si significantly mitigated the adverse effects of low-temperature stress, and thereby plants accumulated lower levels of endogenous ABA. The stress-alleviating impacts of Si by downregulating ABA accumulation were also indicated by the down-regulation of *NCED1* and *PyL-4* expression under low-temperature stress. Stress conditions significantly trigger ABA biosynthesis by regulating β-carotene oxidative cleavage through the activation of the 9-cis-epoxy carotenoid di-oxygenase (NCED) enzyme to break down violaxanthin and neoxanthin to produce xanthoxin and thereby ABA synthesis from its oxidation [57]. Similarly, pyrabactin resistance-like (*PyL-4*) is involved in the regulation of the ABA pathway as an ABA receptor, and it was downregulated in Si treated seedlings, suggesting that Si supplementation had successfully alleviated the detrimental effects of low-temperature stress, and that thereby less ABA was generated in plants. Moreover, an antagonistic trend of JA accumulation with ABA levels was detected in Si supplemented plants under low-temperature stress. However, JA signaling and accumulation in plants has been reported under low-temperature stress conditions for modulating plant responses and tolerance to stress conditions [58]. Under low-temperature stress, JA accumulation can lead to the activation of Inducer of CBF Expression (ICE) proteins via degrading JASMONATE ZIM-DOMAIN (JAZs) proteins, and subsequently regulates the activation of C-repeat-binding factors (CBFs) for inducing the expression of low-temperature-responsive genes [59,60]. Therefore, the higher expression of *ICE1* in Si seedlings under low-temperature stress could be attributed to the Si induced JA accumulation in the current study and consequently aid date palm seedlings in coping with low-temperature stress. Furthermore, date palm seedlings exhibited an increase in endogenous SA level under low-temperature stress. However, Si supplementation further elevated the accumulation of endogenous SA in date palm seedlings in comparison with non Si plants. In contrast, signaling and accumulation of endogenous SA for combating low-temperature stress has been recognized in different plants [61,62]. The current enhancement of endogenous SA in Si date palms is in accordance with the findings of [63], who reported that enhanced endogenous SA levels in cucumber seedlings regulated and induced the expression of low-temperature-responsive genes.

Furthermore, the regulation of plasma membrane ATPase activity under low-temperature stress can lead to the protection of early injury sites and the activation of cellular responses. From the current findings, it is evident that date palm plants exhibited a significant regulation of *PPMA3* and *PPM4.* However, the extent of expression was markedly enhanced in Si treated plants, with an increase in the low-temperature stress level. Previously, [64,65] reported a similar rise in plasma membrane ATPase activity and their transcript levels in Arabidopsis and Cucumber for enhancing tolerance to low-temperature stress. Moreover, an upregulation of *SDR* genes is reportedly involved in the activation of low-temperature-stress responses in plants. The current findings show that the supplementation of Si significantly triggered the transcript accumulation of *SDR* at low-temperature stress (5 °C) in date palms, therefore, suggesting the stress-ameliorative role of Si supplementation. Similar upregulations of SDR genes and proteins are reported for chilling and low-temperature stress in corn and *Medicago truncatula* [66,67]. Moreover, *SRC2* has been described as an activator of the calcium-dependent activation of RBOHF that mediates ROS production and may play a role in low-temperature stress responses [68]. Previously, the expression level of *SRC*2 was studied in a low-temperature-tolerant soybean cultivar (*Glycine max* L. cv. *Kitamusume*), and the transcript accumulation of *SRC2* increased after exposure to 5 °C [69]. Similarly, in the current study, *SRC-2* was upregulated in silicon-treated plants under low-temperature stress and played a critical role in the defense responses of date palm against stress. Such an impact of Si supplementation on date palms led to a positive regulation of date palm physiology by improving growth and providing tolerance against low-temperature-induced hostile conditions. However, further in-depth research is needed at the proteomic and transcriptomic level to investigate the mechanistic aspects of Si interaction with *Phoenix dactylifera* L. for ameliorating low temperature stress induced damages.

## 4. Materials and Methods

### 4.1. Plant Growth under Si Supplementation and Low-Temperature Stress

*Phoenix dactylifera* L. Faradh seedlings (60 days old) were purchased from the Agriculture Research Center in Bahla, Oman. The plants were transferred into pots (10 × 9 cm) containing 250 g of sphagnum peat moss (pH 4.5–5.5, moisture content 38.5%, bulk density 0.7–1.0 mgm^−3^, electrical conductivity 2.0 dSm^−1^, grain size 125–250 μm, organic matter 91.1% *w*/*w*, phosphorus 150–850 mg kg^−1^, sodium (Na) 340 mg kg^−1^), NaCl 850 mg kg^−1^). Before the experiment, the pots with seedlings were shifted to a greenhouse and maintained for one week to acclimatize under optimized growth conditions (relative humidity, temperature, and photoperiod). Daily, 100 mL distilled water was provided to each pot. After that, the seedlings having an equal number of leaves and homogenized length were chosen and arranged in a greenhouse. Silicon solution (Na_2_SiO_3_: 1.0 mM) was prepared in distilled water as it was previously found to have enhanced effects on date palms by our research group [70], and applied (100 mL) to each pot containing seedlings for 28 days. The seedlings (15 plants per treatment) were then divided into three groups and placed at different growth conditions (control: 30 °C; 15 °C and 5 °C) in growth chambers under a randomized experimental design. The seedlings without treatment with silicon (only dH_2_O) were taken as respective controls in each group. To avoid excessive leaching, the silicon solution (100 mL: 1.0 mM) and dH_2_O (100 mL) in relevant pots were applied with caution. The experiment was comprised of the following treatments: (i) control plants treated with only dH_2_O at 30 °C; (ii) Si treated plants at 30 °C; (iii) only 15 °C stress plants; (iv) Si + 15 °C plants; (v) only 5 °C stress plants; (vi) Si + 5 °C plants. After 15 days of exposure to low-temperature stress, the seedlings were harvested in liquid nitrogen and data for various growth parameters were obtained.

### 4.2. Root Phenotypic Data Collection

After harvesting plants, roots samples were collected carefully to avoid root damage or loss. We carefully separated the soil from the roots using fresh water. The clean root samples were transferred to a plastic bag containing distilled water until image analysis to prevent the root from drying. We analyzed the root morphological traits using a 2D image captured by a scanner (Expression 12000XL, Epson, Sakata, Japan). The clean root samples were placed on a transparent tray (30 cm × 20 cm), and tap water was poured carefully until the root samples floated. Images of the floating root samples were captured using the scanner. The root images were added to WinRHIZO 2021 pro software (Regent Instruments Inc., Québec, QC, Canada) for root-morphological data analysis. We annotated and analyzed only the root area in the original image to avoid sampling error data.

### 4.3. Determination of Chlorophyll Contents and Carotenoids

The protocol of [71] was followed with minor modifications for analysis. The leaves from date palm seedlings (200 mg) for each treatment were ground in acetone (80%). The mixture was centrifuged at (12,000× *g* for 10 min) and the supernatant was taken. The absorbance was recorded for chlorophyll a, chlorophyll b and carotenoids, respectively, at 663, 645 and 470 nm.

### 4.4. Leaf Relative Water Content (LRWC) of Date Palm Seedlings

The previously established method of [72] was used to measure the relative water content of date palm seedlings. Fresh samples of leaves were taken for recording fresh mass (Fm). The samples were then kept in a petri dish with 30 mL of distilled water for 6 h to assess their turgid mass (TM). Finally, the leaves were dried in an oven at 80 °C to obtain the dry mass (DM), and the relative water content was calculated using the formula:

RWC [%] = [(FM − DM)/(TM − DM)] × 100.

### 4.5. Quantification of Total Protein Content and Anti-Oxidant Enzymes

For protein determination, the method reported by [2] was followed with slight changes. Briefly, the leaf samples were ground in potassium phosphate buffer (100 mM, pH 6.8) with ethylenediaminetetraacetic acid (EDTA: 0.2 M) using a chilled mortar and pestle. The mixture was then centrifuged at 12,000× *g* for 30 min and the supernatant was employed for protein content measurement. Finally, the absorbance of reaction mixtures was recorded at 595 nm using a spectrophotometer.

Moreover, the antioxidant enzymes assays, i.e., CAT, POD, PPO and TPP, were quantified by using the procedures followed by our research group previously [73] with minor changes. Concisely, the leaves were ground in liquid nitrogen, and 100 mg powdered samples were mixed with 100 mM phosphate buffer (pH 7.0). The homogenous sample was centrifuged at 10,000× *g* at 4 °C for 30 min. The obtained supernatant was used for further analysis.

To determine the POD activity, 100 µL of sample extract with (0.1 M) potassium phosphate buffer (pH 6.8), 50 µL H_2_O_2_ (50 mM), and 50 µL pyrogallol (50 mM) was used to prepare the reaction mixture. The reaction mixture was incubated for 5 min at 25 °C, followed by adding H_2_SO_4_ (50% *v*/*v*) in order to halt the enzymatic reaction. The absorbance of the reaction mixture was measured at 420 nm. By measuring the absorbance value at 420 nm, PPO activity was determined using a reaction mixture identical to that for POD activity but without H_2_O_2_. An increase of 0.1 units of absorbance was used to directly compute a single unit of PPO and POD. For the determination of CAT activity, the protocol described by [73] was employed. Briefly, the sample extract was mixed with H_2_O_2_ (0.2 M) in 10 mM phosphate buffer (pH 7.0). After that, the activity was measured as a reduction in absorbance at 240 nm and reported as units (one unit of CAT was defined as the ng of H_2_O_2_ released/mg protein/min).

### 4.6. Measurement of Lipid Peroxidation

The protocol of [74] was used as reported by [75] to investigate the accumulation of MDA for measuring the level of lipid peroxidation in date palm leaves, with slight modifications. The leaves samples were ground in liquid nitrogen and (100 mg) powder was mixed with 10 mM phosphate buffer (pH 7.0). The reaction sample was processed for centrifugation at 12,000× *g* for 15 min and the obtained supernatant (0.2 mL) was mixed with 0.2 mL of 8.1% sodium dodecyl sulfate (SDS), 1.5 mL of 20% acetic acid (pH 3.5), and 1.5 mL of 0.8% thiobarbituric aqueous acid (TBA) solution in a reaction tube. The reaction mixture was then heated at boiling temperature in a water bath for 60 min. After cooling to room temperature, thereafter, 5.0 mL butanol:pyridine (15:1 *v*/*v*) solution was added to the reaction mixture after it had cooled to room temperature. The top organic layer was removed, and a spectrophotometer was used to measure the optical density of the pink solution at 532 nm.

### 4.7. Superoxide Anion (O_2_^•−^) Activity

The method of [76] was used to estimate the level of O_2_^•−^ in plants, with minor modifications. Briefly, the reaction mixture was prepared by adding powdered plant sample (1 g) to 10 mM phosphate buffer (pH 7.0) containing nitrobluetetrazolium (NBT: 0.05% *w*/*v*) and sodium azide (NaN3: 10 mM) and incubated at room temperature for 1 h. After that, the reaction mixture (5 mL) was transferred into another tube and heated for 15–20 min at 85 ºC. Thereafter, it was cooled down to room temperature and vacuum-filtered. Finally, a spectrophotometer was used to detect the absorbance at 580 nm. The experiment was performed in triplicate.

### 4.8. Elemental Uptake and Accumulation in Date Palm Seedlings

The elemental analysis was measured by adopting the method reported by [75]. Briefly, 1.0 g of soil, roots, and leaf samples were collected to screen for and determine the content of the elements K, Ca, Mg, and Si. Then, 4 mL of Nitric acid (65%) was added to the samples and digested within ultra-microwave, which was then diluted with water to make the final amount 50 mL. The calibration curves were constructed using the appropriate standards and the reaction mixtures were screened with ICP–MS (Optima 7900DV, PerkinElmer, Waltham, MA, USA) as reported in Bilal et al. [76].

### 4.9. SEM analysis of Date Palm Shoot and Root

The procedure established by [76,77] was followed for scanning electron microscopy of samples. Several regions of the fresh shoot and root samples from all experimental groups were taken and kept immediately in Formalin Acetic acid (FAA) fixative buffer. The samples were then dissected under a stereoscope and washed twice with sodium cacodylate buffer (20 min), followed by secondary fixation with osmium tetroxide (OsO_4_: 1%) for 1 h. After that, the samples were rinsed with dH_2_O 2 times and passed through an increasing series of alcoholic concentrations (ethanol: 30%, 50%, 70%, 90% and 100%, 3 times each) to dehydrate the samples fully. The samples were then dried using a critical point dryer, and ethanol was displaced with cold liquid CO_2_. The dried samples were then mounted on an aluminum stub (10 × 10 mm) and coated with plutonium for 3 min. After that, a scanning electron microscope was used to examine each of the mounted samples. The amount of arsenic and silicon in root and shoot samples was then estimated using SEM-EDS (JSM-6490LV, JEOL Ltd., Tokyo, Japan). To determine the atomic percentages of Si or any other elements present in samples, the particulate regions of SEM pictures were examined using EDS.

### 4.10. Phytohormones Extraction and Quantification

The methods of [78,79] were followed to extract and quantify endogenous ABA. In brief, samples from all treatments were freeze-dried and then subjected to gas chromatography mass spectrometry (6890 N network GC system, and 5973 network mass selective detector; Agilent Technologies, Palo Alto, CA, USA) with ABA [(±)-3,5,5,7,7,7-d_6_] ( Cayman Chemical, 1180, Ann Arbor, Michigan 48108 USA) supplemented as internal standard. The spectra recorded in selected ion mode at *m/z* 162 and 190 for Me–ABA and *m*/*z* 166 and 194 for Me–[^2^H^6^]–ABA. The endogenous peaks were compared with corresponding standards and ABA was calculated. The optimized protocol reported previously [80] was followed with minor alterations for extracting and subsequent quantification of endogenous JA. The fragment ion (*m*/*z* 83) was interpreted relatively with JA base peaks and [9,10-^2^H_2_]-9,10-dihydro-JA. Finally, the concentration of JA was estimated by correlating the corresponding peaks and of endogenous JA and respective standards. In a similar manner, Salicylic acid (SA) was extracted using powdered samples of date palm plants by employing the method reported by [78] with slight changes. Briefly, the high-performance liquid chromatography (HPLC) was carried out using a C18 reverse-phase HPLC column (HP Hypersil ODS, particle size 5 µm, pore size 120 Å, Waters, Milford, MA, USA) and a Shimadzu device equipped with a fluorescence indicator (Shimadzu RF-10AxL; Shimadzu, Kyoto, Japan), excitation at 305 nm and emission at 365 nm. The flow was kept constant at 1 mL min^−1^.

### 4.11. Organic Acid Quantification

For extraction and determination of organic acid levels (citric acid, succinic acid and acetic acid) in date palm samples, the protocol reported by [81] was adopted with slight changes. Briefly, the plant samples were freeze-dried and ground to make fine powder.

The samples were then injected for HPLC analysis using a Shimadzu equipment equipped with a fluorescence detector (Shimadzu RF-10AxL) and a C18 reverse-phase HPLC column (HP Hypersil ODS, particle size 5 m, pore size 10; Waters). A flow rate of 0.6 mL min^–1^ was adjusted, and excitation and emission wavelengths were 305 and 365 nm, respectively.

### 4.12. RNA extraction and Quantification

The method developed by [82] was used for RNA extraction from date palm shoots as previously described by [43]. RNA samples were passed through DNase treatment by adding 1 µL DNase I buffer and 1 µL DNase I (2 U), for 10 min at 37 °C to remove gDNA by using Ambion DNase I (RNase free), bovine pancreas 31-kDa glycoprotein (Thermo Fisher Scientific, Waltham, MA, USA). Then, the RNA quality was evaluated on agarose gel electrophoresis and the quantity was measured using the Qubit 3.0 RNA broad-range kit.

### 4.13. cDNA Synthesis and qRT-PCR

For cDNA synthesis, 10 μL (>100 ng μL^−1^) of extracted RNA sample was used. Master Mix was initially prepared by mixing RT buffer (2 μL), 25× dNTPs (0.8 μL), random primers (2 μL), reverse transcriptase (1 μL) and nuclease-free water (3.2 μL). The RNA (10 μL of respective concentration range > 100 ng μL^−1^) was added with Master Mix in PCR tubes. The PCR reaction was run using a thermocycler with specified conditions (25 °C for 10 min, 37 °C for 2 h and 85 °C for 5 min). After completion of reaction, the cDNA was stored at −80 °C.

The cDNA synthesized was employed to amplify the genes (Supplementary Table S1). Actin and Ubiquitin genes were applied as reference genes. Additionally, Power SYBR Green Master Mix and primers (forward and reverse, 10 pM) for each of the target genes were used in qRT-PCR reactions using thermocyclers. The PCR conditions were adjusted as initially at 94 °C for 10 min, followed by 35 cycles at 94 °C for 45 s, 65 °C for 45 s and 72 °C for 1 min, and the extension step was carried out for 10 min at 72 °C.

### 4.14. Statistical Analysis

For analyzing data and designing all graphs, GraphPad Prism was used f (9.5.1; San Diego, CA USA). All data are presented as mean ± SE. Mean values were evaluated using Duncan’s multiple range tests with a significant difference among all eight treatments of the study by ANOVA using SAS software (V9.1, Cary, NC, USA) to find out significant or non-significant treatments by maintaining *p* < 0.05 and, displayed with different lower-case letters. Moreover, *p* < 0.05 between two relevant treatments is flagged with one star (*), *p* < 0.01 is flagged with two stars (**), *p* < 0.001 is flagged as three stars (***) and *p* < 0.0001 is flagged as four stars (****).

## 5. Conclusions

Exogenously applied silicon to date palms resulted in a remarkable induction of low-temperature stress tolerance by enhancing photosynthetic components and water content. Overall, our data predicted the positive effects of silicon, directly stimulating the antioxidant system and indirectly reducing reactive oxygen species. The interaction of silicon with date palm under low-temperature stress can be further correlated with the translocation and accumulation of silicon, resulting in mechanical support to the plant and maintaining nutrient homeostasis for hormonal regulation to modulate the antioxidant defense responses. Moreover, the modulation of stomatal regulation and balanced organic acid led to normal metabolic activities in date palms under low-temperature stress. Likewise, the negative impacts of low-temperature stress were further mitigated by triggering the expression of low temperature stress related genes i.e., *SRC-2*, *ICE1* and the down-regulation of ABA-signaling-related *NCED-1*, *PyL-4.* Further studies are suggested to explore the application of Si in long-term field trials with the advent of global climatic changes as a cost-effective and eco-friendly strategy to overcome low-temperature-induced stress in date palm plants. 

## Data Availability

Not applicable.

## Supplementary Materials

The following supporting information can be downloaded at: https://www.mdpi.com/article/10.3390/ijms24076036/s1.
