# Supplementary material for: Silicon-Induced Morphological, Biochemical and Molecular Regulation in Phoenix dactylifera L. under Low-Temperature Stress
Source: Int J Mol Sci. 2023 Mar 23;24(7):6036. doi: 10.3390/ijms24076036 (PMC10094002; doi:10.3390/ijms24076036)
Supplement: Supplementary file 1 [file ijms-24-06036-s001.zip › ijms-2257019-supplementary.pdf]

Table S1. Date palm primers list

Gene Name	Description	Primer Sequence (5'-3')	Accession Number
LSi-1	Low silicon 1 related gene Oryza sativa Japonica Group aquaporin NIP2-1-like	**F:** GACCTGTCTCGCATATCGCA<br>**R:** TCCAGGGGAAATGCCTGAAC	XM_015770687.1
SOD	Superoxide dismutase [Fe], chloroplastic	**F:** GTTTGGGATTACTCGCCCCT<br>**R:** GCATGCTGCACAAACAGAAC	XM_008807700.3
NCED1	9-cis-epoxycarotenoid dioxygenase	**F:** ACCTACTTCCGCTTTGACCG<br>**R:** TGGGCTTCATGACGATCTGG	XM_008799733.3
SDR	Phoenix dactylifera short chain dehydrogenase/reductase	**F:** ATGTCTAAGCAGAGGTTGG<br>**R:** CTTGTACTTGCACTTGTCCG	XM_008812671.4
PYL4	abscisic acid receptor PYL4-like	**F:** ATGCCTCACCCCGCT<br>**R:** CACCATCGAGCAGCATTGG	XM_008801643.3
PPMA-3	Phoenix dactylifera plasma membrane ATPase-3	**F:** GGATTTCTGCTCACTCCCACT<br>**R:** CTCCAAGGGTATGTTCTCCAAA	XM_039117314.1
PPMA-4	Phoenix dactylifera plasma membrane ATPase-4	**F:** GGAGGTTGCAAGGCTCCGT<br>**R:** CCTCAACATCCATCGCCCAT	XM_008783611.4
ICE1-like	Phoenix dactylifera transcription factor ICE1-like, transcript variant X1	**F:** AAGTCAACCACCCAGGACCA<br>**R:** CGTGATGGGCTTTGCACTTC	XM_008792480.4
SRC-2	BON1-associated protein 2-like	**F:** AGAAGCTCAAGGTGGCTCTG<br>**R:** AGCCTGTAGCTAAGGAAGTGC	XM_039115615.1
Act	Phoenix dactylifera actin	**F:** ATTCTGCAGGACTGGGAAGC<br>**R:** CGAACAGGTTGGCTGAATTGT	XM_008800105.4
